# Race-based differences in serum biomarkers for cancer-associated cachexia in a diverse cohort of patients with pancreatic ductal adenocarcinoma

**DOI:** 10.21203/rs.3.rs-5690506/v1

**Published:** 2025-02-10

**Authors:** Jennifer Permuth, Margaret Park, Evan Davis, Solomon Alhassan, J. Arnoletti, Toni Basinski, Ashley McKee, Mark Bloomston, Tiffany Carson, Tiago Biachi de Castria, Dung-Tsa Chen, Elena Cortizas, Sylvia Crowder, Maria Genilo Delgado, Wade Douglas, Jason Fleming, Pamela Hodul, Kevin Huguet, Kun Jiang, Dae Won Kim, John Koomen, Anjuli Luthra, Mokenge Malafa, Anjana Menon, Raiza Morales, Nipun Merchant, Kenneth Meredith, Qianxing Mo, Manual Molina-Vega, Lina Moreno-Urazan, Kayode Olumoyin, Nathan Parker, Jose Pimiento, Ghulam Rasool, Katarzyna Rejniak, Samer Sansil, Lauren Sparks, Paul Stewart, Alexandra Tassielli, Jamie Teer, Dan Viet Tran, Jose Trevino, Vic Velanovich, Christopher Whelan, Daniel Jeong, Sarah Judge, Andrew Judge

**Affiliations:** H. Lee Moffitt Cancer Center and Research Institute; H. Lee Moffitt Cancer Center and Research Institute; H. Lee Moffitt Cancer Center and Research Institute; H. Lee Moffitt Cancer Center and Research Institute; Orlando Health Cancer Institute; H. Lee Moffitt Cancer Center and Research Institute; H. Lee Moffitt Cancer Center and Research Institute; Ohio State University; H. Lee Moffitt Cancer Center and Research Institute; H. Lee Moffitt Cancer Center and Research Institute; Moffitt Cancer Center; University of Miami; H. Lee Moffitt Cancer Center and Research Institute; H. Lee Moffitt Cancer Center and Research Institute; Florida State University College of Medicine; University of Texas Southwestern Medical Center; H. Lee Moffitt Cancer Center and Research Institute; St. Anthony’s Hospital; Moffitt Cancer Center; H. Lee Moffitt Cancer Center and Research Institute; Moffitt Cancer Center; H. Lee Moffitt Cancer Center and Research Institute; H. Lee Moffitt Cancer Center and Research Institute; H. Lee Moffitt Cancer Center and Research Institute; H. Lee Moffitt Cancer Center and Research Institute; University of Miami; Sarasota Memorial Hospital; H. Lee Moffitt Cancer Center and Research Institute; Lakeland Heath Regional Hospital; H. Lee Moffitt Cancer Center and Research Institute; H. Lee Moffitt Cancer Center and Research Institute; Moffitt Cancer Center; H. Lee Moffitt Cancer Center and Research Institute; H. Lee Moffitt Cancer Center and Research Institute; H. Lee Moffitt Cancer Center and Research Institute; H. Lee Moffitt Cancer Center and Research Institute; Translational Research Institute, AdventHealth; Moffitt Cancer Center; H. Lee Moffitt Cancer Center & Research Institute; Department of Biostatistics and Bioinformatics, Moffitt Cancer Center; University of Miami Miller School of Medicine; Virginia Commonwealth University; University of South Florida; Moffitt Cancer Center; Department of Radiology, Moffitt Cancer Center; Department of Physical Therapy, College of Public Health and Health Professions, University of Florida; Department of Physical Therapy, College of Public Health and Health Professions, University of Florida

## Abstract

Pancreatic ductal adenocarcinoma is projected to become the second leading cause of cancer-related deaths by 2040, with the highest disease burden expected amongst Non-Hispanic Blacks. One of the most significant predictors of poor outcomes is the presence of cancer-associated cachexia (CCa). Yet, race- and ethnicity-specific biomarkers for early CCa diagnosis are lacking. Thus, evaluated a panel of candidate biomarkers of CCa in a diverse cohort of pre-treatment serum. Our study shows that GDF-15 was associated with cachexia severity, was superior to standard CCa-associated biomarkers at classifying cachexia, and differentiated between non-cachexia and pre-cachexia status, but only among Hispanic/Latinx and non-Hispanic Whites. Furthermore, high GDF-15 levels at diagnosis were associated with a ~ 2-fold increase in weight loss over the 6 months post-diagnosis. Thus, GDF-15 may be a potential biomarker for pre-cachexia (prior to weight loss) in the White and the Hispanic population, but not Black individuals. These findings underscore the fact that enrollment of minority individuals in clinical trials to evaluate treatments for CCa is of utmost importance.

## Introduction

Pancreatic ductal adenocarcinoma (PDAC) is increasing in incidence in the United States (US) and globally and is projected to be the second leading cause of cancer-related deaths in the US by 2040^1^. Up to 80% of PDAC cases develop cancer-associated cachexia (CCa), defined as a multifactorial wasting disorder characterized by loss of appetite, body weight, and/or lean body mass (i.e., skeletal muscle), leading to fatigue, functional impairment, treatment-related toxicities, poor quality of life, and reduced survival ^2–5^. Properly detecting CCa in its earliest stage and intervening to maintain muscle mass remain key goals in the care of patients with PDAC. Unfortunately, due to the lack of a well-established definition of CCa and heterogeneity of presentation, diagnosis of CCa typically does not occur until late in the disease course ^6–9^.

Blood-based biomarkers of CCa have the potential to aid in earlier diagnosis and in monitoring the effects of therapy. Laboratory values obtained as part of standard of care shown to have prognostic value for patients with CCa include serum C-reactive protein (CRP), albumin, hemoglobin (HgB), white blood cell (WBC) count, CRP:albumin ratio or the Glasgow Prognostic Score, neutrophil to lymphocyte ratio, platelet count, and bilirubin^10–18^. Human studies conducted among patients with PDAC have also identified candidate circulating biomarkers of CCa which include pro-inflammatory cytokines such as interleukin-6 (IL-6), interleukin-8 (IL-8), tumor necrosis factor (TNF)-α, and monocyte chemoattractant protein-1 (MCP-1), transforming growth factor (TGF)-beta family members such as activin A and growth/differentiation factor (GDF)-15, growth factors including insulin-like growth factor binding protein 2 (IGFBP2), adipokines such as leptin and adiponectin, glycoproteins such as zinc-α2-glycoprotein (ZAG), apolipoproteins including apoC-II, apoC-III, and glucagon-like-peptide-1 (GLP-1), branched chain amino acids, ceramides (medium- or long-chain), and miRNAs^19–26^. Sample sizes of PDAC cases in the aforementioned studies of novel blood-based biomarkers ranged from 60–99, with most but not all studies evaluating patients with metastatic disease at the time of blood collection. Because prior associations with blood-based biomarkers were generally made in patients with advanced disease (who oftentimes were undergoing treatment), these associations may be linked to disease progression rather than CCa. Several of these studies were conducted in Asia and may not be racially diverse or generalizable to other racial/ethnic populations^18,23,27^. Of those conducted in the US^21,22,24,28^, all primarily comprised of self-reported Non-Hispanic Whites (NHW), with only two of these studies ^24,28^ including self-reported Non-Hispanic Black (NHB)/African American individuals (n = 7 ^24^ and n = 11 ^28^), and only one study ^28^ including self-reported Hispanic/Latinx (H/L) individuals (n = 5).

Taken together, it is apparent that investigations of serum biomarkers of CCa in larger and more racially and ethnically diverse cohorts of newly-diagnosed PDAC cases are lacking. Given that PDAC incidence and mortality rates are highest in NHB followed by NHW and H/L^29^, it is prudent to focus research efforts on including historically underserved populations in studies of CCa. The goal of the current study was thus to investigate levels of candidate serum biomarkers in a diverse cohort of treatment-naïve PDAC cases and determine their association with CCa status, survival, and health related quality of life (HRQoL) overall and by race and ethnicity. Importantly, we assessed the added value of a race- and ethnicity-specific panel of biomarkers in predicting clinical outcomes above and beyond standard criteria obtained at the point of care.

## Methods

### Study Participants: Study Population:

The Florida Pancreas Collaborative (FPC) is a multi-institutional prospective cohort study and biobanking initiative established to advance PDAC research in racially/ethnically diverse populations ^30^. Participants in the FPC include 500 individuals with a suspected pancreatic mass presenting to one of the participating study sites. FPC participants meeting the following inclusion criteria were included in this analysis: 1) diagnosed with PDAC; 2) had available pre-treatment blood collections; and 3) were able to have their cachexia status ascertained. A total of 202 participants met these criteria (**Figure S1**). Importantly, systematic differences were not observed between characteristics of PDAC cases who did and did not donate blood (data not shown) ^31,30^. This study was approved by the Moffitt Cancer Center Scientific Review Committee (MCC19717, Pro00029598), and Advarra IRB (IRB00000971). All patients provided informed consent for participation^30^.

### Data collection and Clinical Outcomes:

Demographic, clinical, and epidemiologic data was collected from enrolled participants at baseline and at a 6-month follow-up timepoint through: self-administered online- or teleform-based questionnaires; questions administered by the research coordinator at each participating study site; and abstraction from the electronic medical record^30^. Using available data, participants were first categorized using a schema that classifies the cachexia continuum into 4 stages (non-cachectic (NCa), pre-cachectic (PCa, i.e. those with abnormal labs or appetite who have not lost a significant amount of weight), cachectic (Ca), and refractory cachectic (RCa)) based on clinical, laboratory, functional, and nutritional criteria used by Vigano et al^32^. We also used a simplified method for CCa classification based on self-reported weight loss (WL) over the previous 6 months, with cases having >5% WL or an underweight body mass index (BMI) plus WL >2% categorized as cachectic, and those with <=5% WL as non-cachectic^33^). Due to small sample sizes for sub-groups of participants when using the categorization schema by Vigano et al^32^, the simplified dichotomized categorization of CCa based solely on WL was used for statistical analysis when stratifying by race and ethnicity^33^. As described previously^30,31^, HRQoL was assessed for cohort participants using the European Organization for Research and Treatment of Cancer (EORTC) QLQ-C30 instrument^34^.

### Blood collection and processing:

Blood was collected via phlebotomy and processed into serum using previously described methods at baseline/pre-treatment and at follow-up timepoints^30^ and stored at −80°C. **Table S1** lists all candidate biomarkers, the assay performed, and the % of samples falling within the detectable range. Detailed methods for assays performed are found in Supplemental Methods. Mean % coefficient of variation values (CV) (intra-assay variability) for all successful assays are found in **Table S2**, ranging from 1.16% (ENA-78/CXCL5) to 40.02% (Angiotensin II).

### Statistical Analysis:

Associations among categorical variables such as demographic and clinical variables were examined using Pearson chi-squared test or Fisher’s exact test. For continuous variables, differences between two independent groups were examined by two sample t-test or Wilcoxon rank sum test; differences among three or more groups were examined by Analysis of Variance (ANOVA) or Kruskal-Wallis test followed by Tukey’s Honest Standard Differences or Dunn’s test for pairwise comparisons. Benjamini-Hochberg (BH) method was used for correction for multiple comparisons. Overall survival time was calculated in months from the date of diagnosis to the date of last contact or death. Survival functions were estimated by Kaplan-Meier methods and compared by log-rank test. Cox proportional hazards regression (‘survival’ R package) were used to examine associations between survival and multiple variables^35^. Linear or logistic regression models were used to assess relationships between variables of interest such as serum biomarker levels (predictor variables) and response variables such as HRQoL or cachexia status. Concordance probability estimate analysis was performed using the ‘CPE’ R package^36^. Receiver operator characteristic (ROC) analysis was performed using the ‘pROC’ R package^37^.

## Results

### Characteristics of the study population

**Figure 1:**
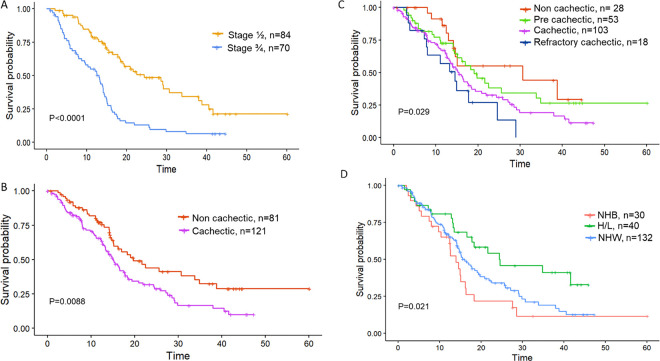
Cachexia status and stage are linked to overall survival in a PDAC cohort with baseline blood available. Survival was analyzed based on cachexia status using either weight loss alone (**A**), cachexia status based on weight loss and other blood biomarkers (**B**) stage (stage 1/2 vs 3/4, **C**) or R/E (**D**). Time to event (x-axis) is measured in months.

Of 500 patients with a suspected pancreatic mass that were recruited to the Florida Pancreas Collaborative study, 318 were confirmed to have a diagnosis of PDAC ^30^. Select sociodemographic and clinical characteristics of PDAC cases who donated blood pre-treatment (n=206) are shown in **Table S3** (stratified by race and ethnicity). The final analytic dataset includes 202 of the 206 PDAC cases with blood for whom cachexia status could be determined using established criteria ^32,33^, and comprised 132 NHW, 30 NHB, and 40 H/L cases (Table 1**, Figure S1**). At enrollment, males were significantly more likely than females to be cachectic (p=0.025). Other predictors of CCa included lower BMI (p=0.006), diabetes (p=0.027), and a worse ECOG functional score (p<0.001) (Table 1). The prevalence of cachexia was highest in H/L (67.5%) followed by NHB (63.3%) and NHW (55.2%). As expected, late-stage disease was also significantly associated with poorer survival (P<0.0001, see Figure 1A). However, cancer stage was *not* associated with cachexia status (Ca vs NCa) via a chi-squared test (P=0.36) or cachexia stage (NCa, PCa, Ca and RCa) via a Cochran-Armitage trend test (p=0.63). Cases classified as Ca (n=121) had significantly worse survival than those classified as NCa (n=81) (log-rank P=0.009, Figure 1B, Table 1, see Figure 1C for survival stratified by cachectic stage as described by Vigano et al^32^).

Compared to NHW, NHB and H/L tended to be diagnosed with PDAC at a younger age and were less likely to have private insurance or to be married. H/L patients in our cohort had a lower overall BMI (p=0.018) than other racial and ethnic groups (**Table S3**). Although NHB presented more frequently with late-stage disease (50.0%) than NHW and H/L (37.5% and 29.4% respectively), NHB did not have significantly poorer overall survival (OS) when compared to the NHW cohort (p= 0.192 based on a pairwise comparison of NHB vs NHW). H/L in our cohort demonstrated a significantly longer OS time than other groups (p=0.021, **Table S3**, Figure 1D).

### Significant differences were observed between cachectic and non-cachectic PDAC cases overall for multiple serum biomarkers.

**Figure 2:**
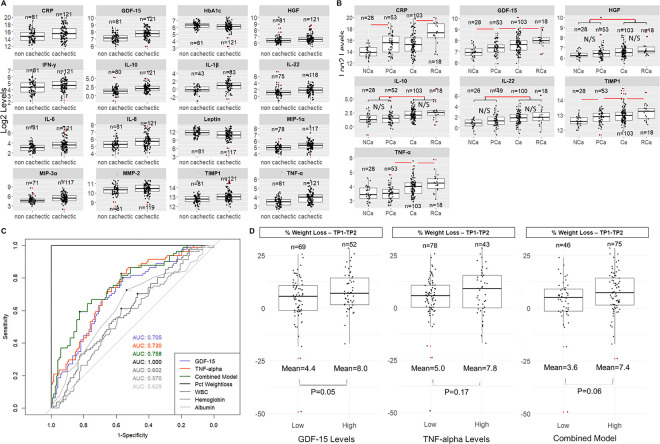
Cachectic and pre-cachectic PDAC patients demonstrate significant differences in stress-related markers at baseline and GDF-15 can be used to classify patients into cachexia categories: Boxplots showing a log-scale value of all significantly different serum biomarkers between cachectic and non-cachectic PDAC patients (**A**) and when stratified into 4 stages as in Vigano et al. (**B**). NCa=non cachectic, PCa=pre cachectic, Ca=cachectic, RCa=refractory cachectic. All plots in **A and B** are significant based on a Wilcoxon rank sum or Kruskal-Wallis test and BH-adjusted p-value. Significant differences in serum biomarkers between groups (pairwise Dunn’s test) are denoted with a red line in **B**. Outliers are denoted by a red dot. **C**) ROC curves for percent self-reported weight loss (Pct Weightloss), GDF-15, TNF-alpha, white blood cell count (WBC), Hemoglobin and Albumin and a combined TNF-alpha * GDF-15 model (Combined Model) using cachexia status at baseline as the “ground truth”. Youden’s optimal thresholds are denoted with a black dot. **D:** Boxplot of percent weight loss (negative values indicate weight gain) from baseline to 6-month follow-up time point. Patients were dichotomized based on levels of GDF-15, TNF-alpha or combined GDF-15 * TNF-alpha levels (Low vs High based on the Youden’s threshold values calculated by the ROC in Figure 2C).

Using hierarchical clustering, biomarker clustering was not observed by CCa status (cachectic versus non-cachectic; **Figure S2**). Among PDAC cases with CCa versus without CCa, serum levels of 16 biomarkers were significantly altered (14 increased, 2 decreased) after adjustment for multiple comparisons, with P_adj_<0.05 (Table 2). TNF-α, GDF-15, IL-10, IL-22, MIP-1α, IL1-β, TIMP-1, IL-8, MIP-3α, IL-6, HGF, IFN-γ CRP, and MMP-2 were significantly higher in patients classified as having CCa compared with those without CCa, whereas leptin and HbA1c were significantly lower in the CCa group (Figure 2A, Table 2). The two most significantly increased markers were TNF-α (~1.4-fold increase) and GDF-15 (~1.4-fold increase), both with an adjusted p-value of <0.001. Separation of patients based on disease stage further revealed three biomarkers that were significantly elevated in cachectic patients with **both** early stage (stage I/II) and late stage (stage III/IV) disease: TNF-α (P=0.014 (early) and P=0.001 (late)), GDF-15 (P=0.003 (early) and P=0.018 (late)), and IL-22 (P=0.004 (early) and P=0.005 (late)), see **Table S4 and Figure S3A**). Among early (but not late) stage PDAC patients, IL-6 (P=0.001), MIP3-α (P=0.002) and IL-8 (P=0.009) were significantly higher in cachectic versus non-cachectic patients (**Table S4 and Figure S3B**).

Patients who rated their health-related quality of life as 3 or less in the EORTC questionnaire at baseline^31^ also displayed significantly higher levels of GDF-15 (P<0.001), TNF-α (P=0.007), TIMP-1 (P=0.009), IL-8 (P<0.001), CRP (P=0.005), and IL-6 (P=0.01).

When stratified by sex, both males and females with Ca demonstrated significant increases in TNF-α, MIP-1α, IL-10, IL-22 and GDF-15 compared to NCa. A decrease in leptin was observed in Ca males and females compared to their non-cachectic counterparts (**Table S5, Figure S4A**). Intriguingly, for several biomarkers, (IL-1b, IL-6, IL-8, TIMP-1, and IFN-g) only females demonstrated significantly increased levels among Ca vs. NCa cases. In contrast, MIP-3α was significantly increased in males with Ca but not females (**Figure S4B**).

### Pre-cachexia stage can be differentiated by GDF-15 and TIMP-1 levels.

When cachexia status was classified using a 4-stage system ^32^, we find that patients with PCa can be differentiated from NCa patients by CRP, TIMP-1 and GDF-15 (Figure 2B, **Table S6**). CRP was omitted from further analysis since it was used to stage cachexia^32^. Other analytes (TNF-α, HGF, IL-10, IL-22) were significantly increased only in patients with Ca or RCa. When TIMP-1 and GDF-15 levels were dichotomized using a median split, both TIMP-1 (P<0.001) and GDF-15 (P<0.001) were significantly increased over worsening cachexia status when subjected to a Cochrane-Armitage trend test. When ordinal regression was performed using cachexia status as the response variable and TIMP-1 and GDF-15 as predictor variables (dichotomized), GDF-15 remains significant as a predictor (P=0.021). Indeed, GDF-15 remains significantly increased over all four cachexia stages using ordinal regression even when adjusting for race and ethnicity, stage, age at diagnosis and BMI (P<0.001, Table 3).

### GDF-15 and TNF-α perform better at cachexia classification than other commonly used classifiers of cachexia.

The area under the curve (AUC), optimal thresholds (using Youden Index), sensitivity and specificity were calculated for all analytes which were significantly different between NCa and Ca patient groups (**Table S7**). These calculations were also performed for other common blood-based biomarkers associated with cachexia status (WBC count, albumin, hemoglobin) ^32^ and for body composition metrics (BMI, waist circumference and waist to hip ratio). Percent WL was used for classification as a (positive) control. Both TNF-α and GDF-15 had AUC values of >0.7 and these analytes were thus used to generate a combined model by multiplying the two concentrations together (Figure 2C). The combined model did have significantly higher AUC than GDF-15 alone (P=0.009) but not better than TNF-α alone (P=0.76); AUCs for TNF-α and GDF-15 were not significantly different (P=0.730). Additional markers did not improve classification (data not shown).

When WL was calculated over time (i.e., from baseline to a 6-month follow-up timepoint), patients with high GDF-15 levels at baseline (based on the optimal ROC threshold) lost a mean of 8.0 pounds and patients with low GDF-15 lost a mean of 4.4 pounds (P=0.05, Figure 2D). TNF-α levels and combined levels of GDF-15 and TNF-α (combined model) did not significantly predict weight loss over the same time period (P=0.17 and P=0.06 for TNF-α and combined values respectively).

### Stratification by race and ethnicity (R&E) reveals novel biomarkers of CCa for NHW and H/L participants.

**Figure 3:**
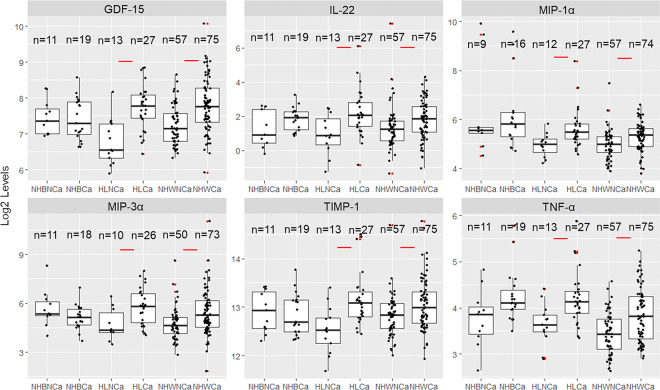
Both NHW and H/L participants demonstrate significant differences between non cachexia and cachexia status in stress-related markers at baseline: Boxplots showing a log-scale value of serum biomarkers which are significantly different between cachectic and non-cachectic PDAC patients for at least two R/Es stratified into Non-Hispanic Black, Hispanic/Latinx and Non-Hispanic White patients. NHB=Non-Hispanic Black, H/L=Hispanic/Latinx, NHW=Non-Hispanic White, NCa=non cachectic, Ca=cachectic. Significant differences in serum biomarkers between groups are denoted with a red line. Outliers are denoted by a red dot.

Of the serum biomarkers significantly deregulated in Ca versus NCa PDAC cases overall (Table 2), several continued to show differences when stratified by R&E. For example, GDF-15, IL-22, MIP-1α, TIMP-1 and TNF-α levels were significantly higher among H/L and NHW PDAC cases categorized as having CCa than those without CCa (Figure 3, **Table S8**). Although statistically significant differences were not observed for any of these biomarkers when comparing NHB with and without CCa (Figure 3, **Table S8**), TNF-α and IL-22 levels did trend upwards for NHB cachectic patients, similar to the other R&E groups (Figure 3). In contrast, although GDF-15 was significantly higher in the cachexia group overall and for H/L and NHW subgroups, this biomarker did not show *any* difference in cachectic NHB compared to non-cachectic NHB PDAC patients (padj=0.95).

### Racial and ethnic variation is apparent for neutrophil- and stress-linked cytokine biomarkers ENA-78/CXCL5 and GRO-α/CXCL1, independent of CCa or pancreas tumor type.

**Figure 4:**
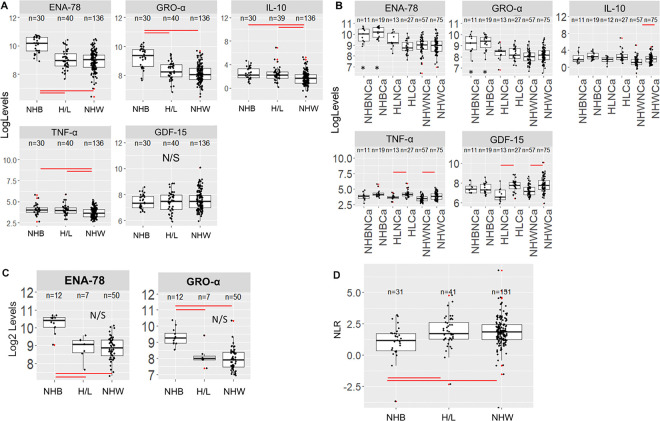
PDAC and other patients demonstrate wide racial variation in select serum biomarkers at baseline: **A**) Boxplots showing a log-scale value of select significant biomarkers when PDAC R&E groups are compared (ENA-78/CXCL5, and GRO-α/CXCL1, IL-10 and TNF-α; GDF-15 was added as a control) and **B**) when stratified into cachexia groups (NHB=Non-Hispanic Black, HL=Hispanic/Latino, NHW=Non-Hispanic White, NCa=non cachexia, Ca=Cachexia). **C**) Boxplots showing ENA-78 and GRO-alpha levels after filtering the full FPC cohort to non-PDAC diagnoses. **D**) Boxplot of neutrophil to lymphocyte ratio (NLR) for PDAC patients in the study. Significant differences between relevant groups are denoted with a red line. Significance between NHBCa/NHBNCa and ***all*** other groups is denoted with a *. N/S = not significant. Outliers are denoted by a red dot.

We also assessed racial and ethnic differences at baseline for the panel of biomarkers studied. A total of 15 biomarkers had significantly different expression levels when comparing NHB, H/L, and NHW PDAC cases in the cohort (n=206) (**Table S9, Figure S5**). 3 of the 13 biomarkers were significantly elevated with a p<0.001 in NHB compared to both NHW and H/L cases and included GRO-α/CXCL1 (P<0.001) and ENA-78/CXCL5 (P<0.001) (**Table S9**, Figure 4A).

Other analytes such as IL-10 (P=0.002) and TNF-α (P=0.002) showed significantly lower levels in NHW compared to other R&E groups (**Table S9**, Figure 4A). The TNF-α/IL-10 ratio^38^ was increased in NHW participants compared to NHB (P=0.02) or H/L (P=0.04) but was not significantly changed for R&E and cachexia status stratification. Other circulating biomarkers that were significantly decreased in NHW compared to the other R&E groups included IL-1β, TNF-α, and TGF-β2.

Further stratification by cachexia status for ENA-78/CXCL5, GRO-α /CXCL1 and GDF-15 (as a comparison) shows that ENA-78/CXCL5 and GRO-α/CXCL1 levels were unchanged for any R&E group when stratified by cachexia status (Figure 4B). When we performed a linear model with either ENA-78/CXCL5 or GRO-α as response variables and R&E, cachexia status, stage, sex, and BMI as predictor variables, NHB demonstrated significantly increased ENA-78/CXCL5 and GRO-α/CXCL1 compared to other R&E groups (**Table S10**). Furthermore, when ENA-78/CXCL5 and GRO-α/CXCL1 levels were compared by R&E in other diagnoses from the FPC cohort (including pancreatic neuroendocrine tumors (PNET), intraductal papillary mucinous neoplasms (IPMN), and pancreatitis), NHB individuals continued to demonstrate significantly elevated baseline levels of these biomarkers compared to other R&E groups (Figure 4C). IL-10 and TNF-α were not significantly changed when non-PDAC individuals were stratified by R&E.

Since both GRO-α/CXCL1 and ENA-78/CXCL5 are linked to neutrophil function, the serum neutrophil to lymphocyte ratio (NLR) was calculated using abstracted clinical data and plotted by R&E. We find that NHB PDAC patients demonstrated a small but significant *decrease* in NLR when compared to H/L and NHW (P<0.001 for both pairwise comparisons) (Figure 4D). Our data indicate that both higher levels in total lymphocyte count and lower levels in neutrophil count in NHB participants are responsible for this measure.

### Survival is predicted by inflammatory and proliferative cytokine levels.

**Figure 5:**
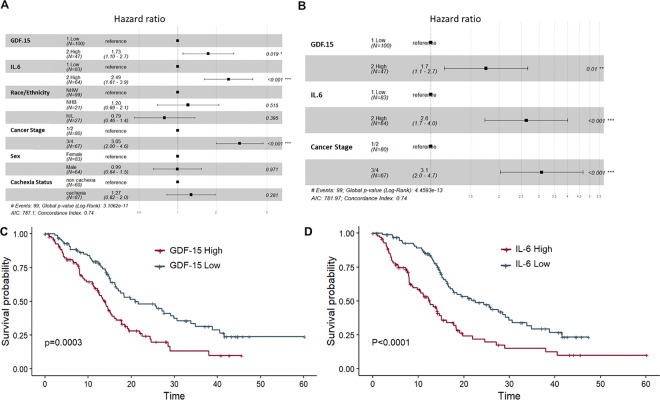
GDF-15 and IL-6 are linked to overall survival both CoxPH models and by Kaplan estimation: **A-B**) Forest plots of dichotomized analytes found to be associates with survival as continuous variables in a univariate analysis when controlled for demographic and patho-clinical variables (**A**). B represents the final model. NHB=Non-Hispanic Black, HL=Hispanic/Latinx, NHW=Non-Hispanic White, NCa=non cachectic, Ca=Cachectic. **C-D**) Median levels of GDF-15 (**C**) or IL-6 (**D**) were calculated and participants were classed into either low (Low) or high (High) groups based on a median split. **C**) and **D**) represent Kaplan-Meier survival curves (time in months) for GDF-15 and IL-6, respectively. Time = time to event in months.

A univariate Cox proportional hazard analysis indicates that, among all biomarkers which were significantly different in Ca vs NCa PDAC patients, GRO-α, CRP, TNF-α, GDF-15, HGF, IL-10, IL-6, IL-8 and TIMP-1 are all independent predictors of OS in the full cohort as continuous variables (**Table S11**). However, after dichotomizing these analytes via a median split and controlling for BMI, R&E, stage, cachexia status, sex, and age at diagnosis (Figure 5A), a modified model including GDF-15, IL-6 and stage as significant predictors of survival was reached (Figure 5B). Furthermore, when dichotomized based on a median split, high GDF-15 and high IL-6 predict shorter survival times via the Kaplan-Meier estimation (Figure 5C–D). To assess added value of GDF-15 for predicting overall survival, a Gonen and Heller concordance probability estimate (CPE)^36,39^ analysis was performed on Coxph models using GDF-15 alone (CPE=0.600, SE=0.022), IL-6 alone (CPE=0.575, SE=0.019), IL-6 and GDF-15 (CPE=0.608, SE=0.021) and GDF-15, IL-6 and WL (CPE=0.617, SE=0.022), all as continuous variables. All differences in calculated CPEs are thus within the standard error. A source file of all analyte levels, neutrophil and lymphocyte counts, longitudinal weight loss, cachexia status, diagnosis and survival data for all 275 patients with baseline blood available for analysis (for patients with any diagnosis) may be found in **Table S12**.

## Discussion

The FPC cohort represents one of the most racially and ethnically diverse group of PDAC patients with CCa status and available serum biomarker data analyzed to date ^18–25,40–44^, and we find that racial disparities in outcomes are in general agreement with published outcomes from others by race or ethnicity^45,46^.

In the current investigation, growth differentiation factor-15 (GDF-15), also known as macrophage inhibitory cytokine 1 (MIC1), was identified as an important marker of *pre*-cachexia which continues to increase through the cachexia continuum, and which predicts survival among male and female H/L and NHW participants. GDF-15 is a pleiotropic molecule involved in the pathophysiology of cancers, cardiac disease, COVID-19, hyperemesis gravidarium and is tightly linked to stress pathways^47–51^. It is known as a key biomarker of stress, dysfunctional metabolism and energy production and has emerged as an attractive therapeutic target for obesity/metabolic disorders, cachexia, and immunotherapy^48,52,53^. Anti-GDF-15 monoclonal antibody treatment is both well tolerated and may reverse anti-PD-L1 resistance in clinical trials^54^ thus this treatment modality may be useful in contexts other than CCa. In contrast to others^26^, we do not observe sexual dimorphism in cachexia-related GDF-15 upregulation^55^. Importantly, this molecule is found to be upregulated in the circulatory system of both early (I/II) and late (III/IV) stage patients, suggesting that the increase in GDF-15 is not secondary to cancer progression. Furthermore, our findings strongly suggest that this molecule should *not* be used as a marker for cachexia in NHB PDAC patients due to the possibility of false negatives. Importantly, GDF-15 was one of only two markers which was predictive of “pre-cachexia” status and this molecule predicted survival independently of weight loss based on both a Kaplan Meier estimate and CPE analysis. Early identification and intervention for cancer-related cachexia is key to alleviating the negative outcomes associated with this condition^5,21,26,32,56,57^. Ponsegromab (a monoclonal GDF-15 antibody) has shown promise in clinical trials^53^ but this trial importantly did not include any NHB individuals. Thus, these findings underscore the importance of minority inclusion in clinical trials but are valuable from an interventional standpoint for H/L and NHW populations.

One intriguing finding which emerged from this study is that, although TNF-α and IL-22 did not reach significance for the smaller NHB group in our cohort, the means did trend upwards for these two markers in cachectic NHB patients, in stark contrast to GDF-15 and TIMP-1. Thus, these molecules may warrant further investigation as racially agnostic indicators of metabolic stress. TNF-α may be of special interest, as this molecule has long been studied as a metabolic stress and inflammatory cytokine^58,59^. Our data demonstrate that NHB and H/L participants have slightly higher circulating TNF-α levels than NHW in line with previously published literature and secondary to increased chronic or acute social stressors minority groups experience that increase inflammation^60,61^. Higher baseline levels of TNF-α in minorities may thus serve to blunt increases in this cytokine related to cachectic stress. Nevertheless, with a larger sample size, increases in this cytokine may become apparent for NHB individuals with cachexia.

The role of GDF-15 and race is understudied. However, some studies include that of Rybicki et al^62^, who observed lower GDF-15 expression in male NHB participants in a benign biopsy prostate cancer risk study. Contrary to these findings, a study in 2012 identified Black race as being predictive of higher plasma GDF-15 levels in a study of atherosclerosis^63^. Hence, this work represents the first time that GDF-15 has been studied in the context of a response to metabolic stress (cachexia) and race. Taken together, these findings suggest that monitoring of weight/weight loss is of paramount importance in the diagnosis of CCa in NHB patients, as biomarkers of CCa for this population remain elusive.

Regarding our stratification based on sex, in general agreement with other literature^55^, we observe sexual dimorphism in circulating analytes for cachexia. Thus, these findings clearly demonstrate the need to include sex as a biological variable in studies of CCa.

Another molecule we found to be predictive of PCa, Ca, and RCa is the tissue inhibitor of metalloproteinases, TIMP-1^64^. TIMP-1 is a pro-inflammatory cytokine associated with and proliferative processes^64^. Although TIMP-1 is not known to be associated with GDF-15 activity in cancer, TIMP-1 and GDF-15 were weakly correlated in a study of cardiovascular disease in aging patients, suggesting a prognostic use for these markers in a fingerprint^65^ to better predict cachexia status.

In contrast to others^21^, we did not observe increases in circulating monocyte chemoattractant protein 1 (MCP-1) in cachectic patients either in the full cohort or when we restricted our analysis to early stage PDAC. However, we did not stratify our cachectic cohort to those who lost > 10% of their body weight as in^21^. MCP-1 was a successful assay in our hands, and thus we are unable to explain this finding as a technical issue. However, we did observe that this analyte was decreased in minority PDAC patients at baseline (**Figure S5**) which may have masked CCa-induced increases observed in our diverse cohort. In general agreement with other studies^40,51,66,67^ however, we do observe increases in circulating GDF-15, TNF-α, IL-6 and decreases in leptin in cachectic PDAC patients.

Other main findings are the distinct racial differences in the TNF-α/IL-10 inflammatory/anti-inflammatory signaling axis^67^ and the aging/inflammatory biomarkers ENA-78/CXCL5 and GRO-α/CXCL1^20,40,68^. ENA-78/CXCL5 and GRO-α/CXCL1 are of particular interest, as they are upregulated in NHB patients *independent of CCa status and pancreas tumor type*. ENA-78/CXCL5 is a member of the CXC chemokine family and is involved in neutrophil signaling and recruitment^69^. Similarly, the chemokine CXCL1 is an angiogenic inflammatory marker which has been linked to neutrophil recruitment and migration of adipose stromal cells into the tumor microenvironment^70^. ENA-78/CXCL5 and GRO-α/CXCL1 have been shown to have increased expression in tumors of patients with malignant versus non-malignant pancreas tumors, suggesting a role for these genes in pancreatic carcinogenesis. ENA-78/CXCL5 has been shown to promote pancreatic cancer cell growth, migration, and invasion, and to predict poor prognosis. Other findings indicate that GRO-α/CXCL1 and ENA-78/CXCL5 are associated with stress due to social disruption, adult adversity and/or psychological comorbidity in chronic pain disorders^71,72^. Moreover, these signaling molecules have been associated with PDAC immune evasion^73,74^, lending credence to a racially unbalanced CXCL1/5-signaling “tumor-friendly” environment. We find that a higher circulating CXCL1/ENA-78/CXCL5 signature is associated with the NHB race, but *not* with higher circulating neutrophils in NHB patients in agreement with published literature^75^, suggesting that this minority group may be desensitized to cytokine-mediated neutrophil mobilization or signaling. They may also suggest that NHB have increased formation of neutrophil extracellular traps either during cancer initiation or progression. The current literature indicates that both incidence and mortality for PDAC are increased in NHB patients compared to Whites in the US^76^, thus the current investigation may shed light on this disparity.

**Figure 6:**
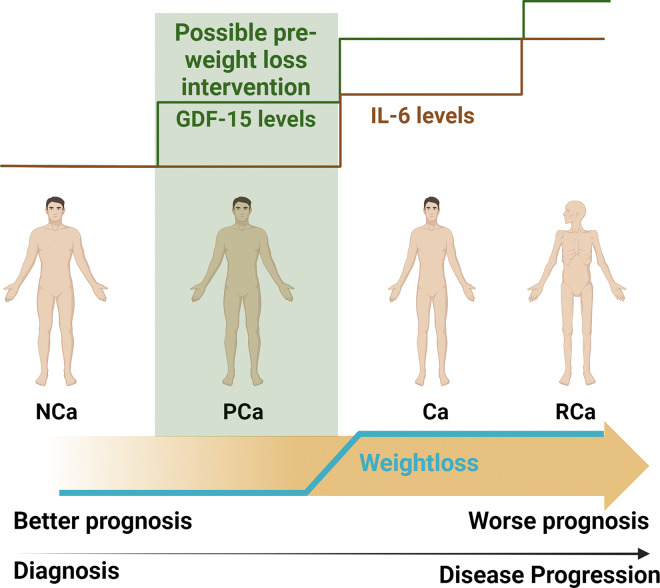
Overview of IL-6 and GDF-15 levels throughout the cancer-associated cachexia continuum.

Interestingly, the cytokine in our panel with the strongest association with overall survival was IL-6. Consistent with other reports, IL-6 was found to be increased only in refractory cachectic patients in our cohort and was *strongly* associated with decreased survival^66,77^. IL-6 remained a significant predictor of overall survival in our large study, even when controlled for multiple covariates, suggesting a renewed interest in this prognostic marker may be warranted^77^. Indeed, for non-NHB patients, our findings suggest that GDF-15 is a marker for *early* cachexia and that IL-6 increases at a *later* stage in the cachectic continuum (i.e. when the patient has refractory disease). Thus, it is possible that a subset of patients with pre-cachexia and elevated GDF-15 do not go on to develop cachexia. Such patients could have significantly better outcomes than those with elevated IL-6 and refractory cachexia. Collectively, our findings suggest that GDF-15, while less predictive of survival outcomes, has utility as an *early* marker of metabolic stress. Thus, there may be a clinical benefit to evaluating GDF-15 levels as soon as possible after a probable PDAC diagnosis is made to alert clinicians of potential future weight loss and supportive care needs necessitating intervention (Fig. 6, created in biorender.com).

Overall, our findings suggest that, whereas non-Hispanic Whites and Hispanic/Latinx elevate circulatory GDF-15 levels in response to cachexia/metabolic stress, NHB do not. Furthermore, there may be an NHB-specific chronic and non-PDAC-influenced ENA-78/CXCL5/GRO-α/CXCL1 inflammatory phenotype for this minority, possibly due to social stress or allostatic load. Indeed, future studies by this group will investigate a possible link between adipose tissue deposition, cytokine signaling, race and PDAC progression.

Our cohort of patients was recruited not only from academic centers, but also from community hospitals in the area^30,45^. As such, this cohort represents a more “real-world” scenario regarding choice of therapy, outcomes and racial and ethnic diversity than many previous cohorts. Nevertheless, a limitation of the study is that none of the participants were “normal” healthy controls because all participants presented with a suspicion of a pancreatic mass. In addition, we note a wide range of mean %CV for our assays, suggesting that some biomarkers may be less amenable to assessment using antibody-based techniques. Nevertheless, all reliably dysregulated biomarkers chosen for further evaluation from our panel had an intra assay variability of < 20%. Finally, although our cohort was more diverse than others including clinical trials of cachexia therapies, there are only 30 NHB PDAC patients with baseline blood available in our cohort. Hence, trials aimed at specifically recruiting minority populations are crucial to fully understanding the nature of racial disparities in CCa and PDAC.

## Conclusions

GDF-15 has emerged as a promising cachexia biomarker and is specifically increased prior to weight loss for *non-NHB* PDAC patients. Furthermore, although GDF-15 did not associate with cachexia status/weight loss in NHB patients, it was associated with survival even when controlled for R&E. Future work should endeavor to ensure that minority participants are well represented in PDAC cohorts so that the true biomarker landscape is evaluated from diverse populations. Furthermore, these studies suggest that sex should be included in any cachexia biomarker evaluation.

We also conclude that the inflammatory cytokine TNF-α warrants further investigation in a larger population of minority PDAC patients as the lack of increase in CCa-related TNF-α may be secondary to higher circulating levels of this cytokine in minority *non-PDAC* participants.

In addition to our GDF-15 findings, we report that the serum cytokine landscape for NHBs is decidedly neutrophil-linked and pro-inflammatory compared to either other minorities (H/L) or NHWs, but that circulating neutrophils are lower in the NHBs. Caution should be taken in interpreting these findings, however, to avoid over-simplifying the complex interplay between genetic background, social stressors, R&E and the metabolic stress response resulting from cancer-associated WL.

## Figures and Tables

**Table 1:** Sociodemographic and clinical characteristics of Florida Pancreas Collaborative participants with known cachexia status and baseline blood^^^ (n=202)*.

		NCa (n=81)	NCa %	Ca (n=121)	Ca %	p-value
**Age at diagnosis, mean (SD)**		68.6 (11.1)		69.0 (10.2)		0.826
**Sex**	Female	52	64.20	57	47.11	**0.025**
	Male	29	35.80	64	52.89	
**Race and Ethnicity**	Non-Hispanic Black (NHB)	11	13.58	19	15.70	0.442
	Hispanic/Latinx (H/L)	13	16.05	27	22.31	
	Non-Hispanic White (NHW)	57	70.37	75	61.98	
**Education**	Post-graduate	19	23.46	10	8.26	**0.003**
	Up to college	29	35.80	31	25.62	
	Up to high school	9	11.11	28	23.14	
	Missing	24	29.63	52	42.98	
**Income**	$0–$60,000	17	20.99	33	27.27	0.298
	$60,000–$100,000	15	18.52	14	11.57	
	Over $100,000	9	11.11	12	9.92	
	Missing	40	49.38	62	51.24	
**Insurance status**	Insured	52	64.20	69	57.02	0.618
	Uninsured	0	0.00	2	1.65	
	Missing	29	35.80	50	41.32	
**Marital Status**	Married	42	51.85	46	38.02	0.138
	Unmarried	12	14.81	26	21.49	
	Missing	27	33.33	49	40.50	
**Stage**	I/II	43	53.09	50	41.32	0.210
	III/IV	26	32.10	44	36.36	
	Missing	12	14.81	27	22.31	
**Grade**	1 (well-diff)	3	3.70	2	1.65	0.759
	2 (moderately diff)	25	30.86	32	26.45	
	3 (poorly diff)	14	17.28	19	15.70	
	4 (undifferentiated)	0	0.00	0	0.00	
	Missing	39	48.15	68	56.20	
**BMI Category**	Underweight	5	6.17	16	13.22	**0.021**
	Normal	20	24.69	42	34.71	
	Overweight	29	35.80	43	35.54	
	Obese	25	30.86	18	14.88	
	Missing	2	2.47	2	1.65	
**BMI, mean (SD)**		27.8 (5.4)		25.7 (5.0)		**0.006**
**Family history of pancreatic cancer**	No / Unknown	72	88.89	119	98.35	0.817
	Yes	9	11.11	2	1.65	
**Smoking status**	Never	31	38.27	36	29.75	0.117
	Former	26	32.10	37	30.58	
	Current	2	2.47	11	9.09	
	Missing	22	27.16	37	30.58	
**Age-adjusted CCI**	0–5	13	10.74	18	22.22	1.000
	>5	33	27.27	50	61.73	
	Missing	35	28.93	53	65.43	
**Diabetes**	Yes	20	24.69	46	38.02	**0.027**
	No	35	43.21	53	43.80	
	Missing	26	32.10	22	18.18	
**% Weight loss, median (SD)**		1.70 (2.8)		11.1 (5.3)		**<0.001**
**Waist circumf, median (SD)**		36.56 (6.2)		37.80 (15.4)		0.731
**Waist-Hip Ratio, median (SD)**		0.92 (0.11)		0.92 (0.07)		0.360
**ECOG functional score**	0	53	65.43	38	31.40	**<0.001**
	1	25	30.86	70	57.85	
	2	1	1.23	6	4.96	
	3–4	0	0.00	3	2.48	
	Missing	2	2.47	4	3.31	
**Survival in months, median (LCI;UCI)**		19.7 (15.1;38.8)		15.1 (13.3;17.8)		**0.009**

Abbreviations: BMI=Body Mass Index; Ca=Cachectic; CCI= Charlson Comorbidity Index; NCa=Non-cachectic; LCI=Lower confidence interval; UCI=Upper confidence interval; SD=standard deviation; circumf = circumference

* 4 NHW patients with baseline blood (out of 206 total participants) could not be assessed for cachexia status due to missing weight values.

^ Cachexia staging according to Fearon et al (39) with the following modification: skeletal mass index (SMI) was not assessed.

**Table 2:** Means and standard deviations for all biomarkers successfully tested, stratified by cancercachexia status and sorted by adjusted P-value*

Analyte	NCa mean (n=81)^^^	Ca mean (n=121)^^^	NCa sd	Ca sd	p (Wilcoxon)	padj (BH)
TNF-α	3.513	3.991	0.492	0.598	<0.001	**<0.001**
GDF-15	7.207	7.713	0.580	0.716	<0.001	**<0.001**
IL-10	1.482	2.236	1.107	1.100	<0.001	**<0.001**
IL-22	1.267	1.905	1.252	1.135	<0.001	**<0.001**
MIP-1α	5.120	5.485	0.943	0.865	<0.001	**<0.001**
IL-1B	0.024	0.999	1.098	1.271	<0.001	**<0.001**
Leptin	11.759	10.786	1.460	1.918	<0.001	**0.002**
TIMP-1	12.783	13.048	0.452	0.526	<0.001	**0.002**
IL-8	5.291	5.713	0.610	0.866	0.001	**0.002**
MIP-3α	4.896	5.481	1.079	1.271	0.001	**0.002**
IL-6	3.248	3.725	0.892	1.061	0.001	**0.003**
HbA1c	6.307	6.109	0.425	0.581	0.004	**0.016**
HGF	6.502	6.687	0.680	0.616	0.005	**0.016**
IFN-γ	4.330	4.700	0.992	0.864	0.005	**0.016**
CRP	14.927	15.623	1.611	1.972	0.014	**0.038**
MMP-2	10.273	10.436	0.565	0.523	0.019	**0.050**
Albumin	26.314	26.237	0.258	0.223	0.040	0.099
TGF-β2	4.689	4.871	0.798	0.904	0.050	0.116
Glucose	4.519	4.632	0.410	0.363	0.058	0.129
HDL	6.415	6.311	0.536	0.499	0.124	0.245
GRO- α	8.223	8.378	0.749	0.735	0.128	0.245
LDL	10.081	10.198	0.573	0.635	0.128	0.245
CCK	5.841	5.759	0.512	0.557	0.171	0.312
a-SMA	0.744	0.707	0.577	0.676	0.310	0.542
PPAR-γ	1.168	1.253	0.663	0.643	0.325	0.545
C peptide	9.797	9.838	0.747	0.757	0.380	0.614
ENA-78	9.127	9.109	0.811	0.802	0.581	0.875
MCP-1	7.925	7.960	0.370	0.437	0.583	0.875
Adiponectin	16.692	16.687	0.610	0.524	0.612	0.886
Activin A	6.900	6.985	1.396	1.298	0.677	0.908
Lumican	14.512	14.525	0.465	0.459	0.716	0.908
Triglyceride	3.545	3.573	0.546	0.556	0.717	0.908
Fibronectin	18.872	18.909	0.893	0.852	0.724	0.908
TGF- β1	11.020	11.054	0.413	0.434	0.751	0.908
HIF-1α	6.550	6.480	1.200	1.306	0.793	0.908
Insulin	4.076	4.043	1.060	0.891	0.830	0.908
Angiotensin II	−0.218	0.023	1.951	2.427	0.833	0.908
G.CSF	4.794	4.864	0.629	0.831	0.842	0.908
Laminin	7.528	7.529	0.390	0.491	0.869	0.908
ZAG	15.379	15.355	0.383	0.457	0.871	0.908
MDC	9.399	9.373	0.338	0.415	0.887	0.908
CA19–9	3.371	3.227	2.158	2.458	0.949	0.949

* All values are Log2 transformed.

^ Number of samples for analytes for which 100% of samples tested were in range.

**Abbreviations:** BH=Benjamini-Hochberg; Ca=Cachectic; NCa=Non-cachectic; p=p-value; padj =adjusted p-value; sd=standard deviation.

**Table 3:** High GDF-15 expression is associated with pancreatic cancer-associated cachexia stage*

Characteristic	OR	95% CI	p-value (global)
High GDF-15	4.01	2.38, 6.97	<0.001
Race/Ethnicity			0.12
NHW	–	–	
NHB	2.45	0.93, 6.68	
H/L	1.87	0.75, 4.80	
Age at diagnosis	0.99	0.96, 1.02	0.5
BMI			0.2
Normal	–	–	
Underweight	2.18	0.65, 7.46	
Overweight	0.81	0.36, 1.81	
Obese	0.58	0.24, 1.39	
Stage			0.11
1/2	–	–	
3/4	1.68	0.88, 3.24	

Abbreviations: OR=Odds ratio; CI=Confidence interval; NHW=Non-Hispanic white; NHB=Non-Hispanic black; H/L=Hispanic/Latinx; BMI=Body mass index.

* Patients were separated into two groups based on their GDF-15 levels (via a median split). Ordinal regression was performed using cachexia stage (based on the Vigano et al classification (32)) as the response variable and GDF-15, race/ethnicity, age, BMI or stage as predictor variables.
